# Microbiologically Influenced Corrosion of Aerospace-Grade Aluminum by SRB-Enriched Biofilms Isolated from the Mars Analog Lake Salda

**DOI:** 10.3390/microorganisms13112555

**Published:** 2025-11-08

**Authors:** Tuba Unsal, Seben Yucel, Demet Ongan Rabba, Abdullah Aksu, Omer Suat Taskin, Mehmet Emre Cetintasoglu, Rasit Bilgin, Nagihan Korkmaz, Esra Billur Balcıoglu Ilhan, Osman Dur, Nuray Caglar Balkis

**Affiliations:** 1 Institute of Marine Science and Management, Istanbul University, Vefa, 34134 Istanbul, Türkiye; sebenyucel@istanbul.edu.tr (S.Y.); ongan@istanbul.edu.tr (D.O.R.); aaksu@istanbul.edu.tr (A.A.); omert@istanbul.edu.tr (O.S.T.); emre.cetintasoglu@istanbul.edu.tr (M.E.C.); nagihan.ersoy@istanbul.edu.tr (N.K.); ebillur@istanbul.edu.tr (E.B.B.I.); osmandur@istanbul.edu.tr (O.D.); nbal@istanbul.edu.tr (N.C.B.); 2 Institute of Environmental Sciences, Boğaziçi University, Bebek, 34342 Istanbul, Türkiye; rasit.bilgin@bogazici.edu.tr

**Keywords:** microbiologically influenced corrosion (MIC), Lake Salda, Mars

## Abstract

Lake Salda in Türkiye serves as a valuable Earth analog for studies of the properties of Mars due to its mineralogical and microbiological similarities to Jezero Crater on Mars. This study investigated the role of sulfate-reducing bacteria (SRB) enrichment culture isolated from Lake Salda on the microbiologically influenced corrosion (MIC) of an aluminum alloy (AA7075) using electrochemical, microbiological, molecular, and spectroscopic methods. Potentiodynamic polarization (PDP) tests confirmed SRB-enriched biofilm significantly accelerated corrosion. Fourier Transformed Infrared Spectroscopy (FTIR) further distinguished the control and biotic surfaces, showing the replacement of a 980 cm^−1^ polysaccharide band with a 1075 cm^−1^ cyclic polysaccharide vibration in SRB-colonized coupons. This spectral transition reflects biofilm maturation and EPS accumulation, providing molecular evidence for SRB-driven MIC. Molecular analysis identified Proteobacteria and Firmicutes as dominant phyla, and *Desulfofustis limnaeus* was detected in Lake Salda for the first time. Moreover, benthic foraminifera and ostracods were observed, some with morphological anomalies. These results provide mechanistic insight into the biochemical and electrochemical interactions driving SRB-induced corrosion, highlighting Lake Salda’s importance for studying microbial–material interactions in extreme environments.

## 1. Introduction

Approximately 4.5 billion years ago, voluminous volcanic activity led to the substantial release of gases into Earth’s primordial atmosphere [1]. The predominant gas during this period was water vapor, accompanied by other gaseous constituents such as carbon dioxide, hydrogen chloride, molecular hydrogen, hydrogen sulfide, sulfur dioxide, hydrogen fluoride, carbon monoxide, and helium [2]. Sulfur and sulfur compounds, including sulfides, bisulfides, thiosulfates, hydrogen sulfide (H_2_S), polythionates, and sulfuric acid, played a key role in shaping early biogeochemical cycles and provided electron acceptors for anaerobic microbial metabolism [3]. Sulfate-reducing bacteria (SRB), capable of utilizing sulfate as a terminal electron acceptor, are considered among the earliest life forms to influence redox reactions and metal corrosion in anoxic environments. Understanding these microbial sulfur processes is essential to unraveling the mechanisms behind microbiologically influenced corrosion (MIC). The microbial ecology of sulfur-rich environments remains insufficiently understood, largely because of the coexistence of aerobic and anaerobic organisms and the mutualistic and successional interactions between heterotrophs and autotrophs Moreover, the physical scale over which sulfur cycling influences corrosion varies significantly depending on specific environmental settings [3].

Mars is considered one of the most promising candidates in the solar system for the potential preservation of biosignatures due to its past water activity and sedimentary ge-ology. In 2024, researchers analyzing seismic data from NASA’s InSight Mars lander pro-posed the existence of a subsurface reservoir of liquid water located approximately 10–20 km beneath the Martian crust [4]. Lake Salda in Türkiye has been suggested as a relevant Earth-based environment for understanding how biosignatures may form under extreme alkaline conditions. Although the biological and atmospheric characteristics of Mars and Earth differ significantly, both Jezero Crater and Lake Salda share similarities in their mineralogical composition, including the presence of carbonates and magnesium-rich clays, both minerals of astrobiological interest. Thus, rather than serving as a direct analog, Lake Salda functions as a valuable testbed for studying microbial–mineral interactions that may inform future Mars exploration strategies.

Lake Salda is a closed alkaline water body (pH > 8.4) and the height of the lake surface above sea level is around 1140 m. It has a surface area of ca. 45 km^2^ and an average depth of 80 m and a maximum of 200 m [5]. This lake’s composite stromatolites are formed by the microbially induced precipitation of hydromagnesite [6]. The fossil stromatolites found in Lake Salda prove the presence of algae, diatoms, and cyanobacteria. However, some studies have reported that cyanobacteria may coexist with SRB [7]. Benton Clark III, a Mars Exploration Rover (MER) science team member says that the most popular candidates for life on Mars are members of the *Desulfovibrio* genus [8]. Recent analyses of samples from the Bright Angel Formation by the Perseverance rover have identified minerals such as vivianite (Fe^2+^-phosphate) and greigite (Fe-sulfide), which commonly form under anoxic, organic-rich conditions on Earth, often mediated by SRB. While the origin of these minerals on Mars remains unresolved, whether biotic or abiotic, their presence strengthens the case for exploring biosignatures in extreme Earth environments. These findings do not provide definitive evidence of past Martian life but offer significant clues regarding the planet’s ancient habitability. Continued in situ research and future sample return missions will be critical in uncovering the processes behind these mineral formations and their implications for life detection on Mars [9].

SRB are the most well-known and most blamed group for causing MIC. These bacteria are chemoautotrophs, deriving their organic nutrients through chemical reactions, with some species capable of fixing inorganic carbon from CO_2_ to produce organic matter. In 2019, NASA published a report called “Corrosion on Mars” which examined environmental factors affecting the corrosion of spacecraft materials in Martian conditions [10,11]. The report also documented structural deterioration observed on the wheels of the Curiosity rover, attributing these damages to corrosive interactions with transient liquid brines reportedly present on Mars. Although these findings do not directly prove the presence of corrosion caused by microbial activity, they underscore the importance of investigating corrosion processes that may occur under extreme conditions. In this context, Lake Salda, characterized by its high alkalinity and harsh environmental parameters, serves as a valuable natural testbed. Studying microbial and chemical interactions in such extreme environments can contribute to the development of hypotheses regarding potential corrosion mechanisms and biosignature preservation on other planetary bodies.

Aluminum alloys are widely used in the aerospace industry due to their low mass density, high strength, excellent mechanical properties, and high corrosion resistance [12]. The most widely used aluminum alloys in aerospace industries are the Al-Zn alloys (7xxx series), Al-Cu alloys (2xxx series), and Al-Li alloys [13]. AA7075 aluminum alloy, has been used in many spacecraft and aerospace applications such as Apollo spacecraft, the Space Shuttle, and the International Space Station. Under anaerobic conditions some mechanisms were suggested for MIC by SRB [14]. One widely accepted mechanism is the extracellular electron transfer (EET) theory, which explains how SRB acquire electrons from metal surfaces. EET happens in two ways: direct electron transfer (DET) and mediated electron transfer (MET). In the DET mechanism, electrons are transferred directly from the metal to the bacterial cell through outer-membrane cytochromes or conductive pili (nanowires). However, in early biofilm formation, DET is limited due to the presence of a natural oxide layer on the metal surface, which hinders direct contact. During the logarithmic growth phase, SRB secrete extracellular polymeric substances (EPS) and metabolic byproducts, which help disrupt or degrade this passive oxide layer. Once the barrier is compromised, DET becomes more effective, making the metal surface more susceptible to corrosion. In the MET pathway, small redox-active molecules (such as hydrogen, flavins, riboflavin, or quinones) transfer electrons between the metal surface and SRB cells. These mediators not only enhance electron transport but are also implicated in the biogeochemical transformation of sulfur compounds, particularly the stepwise reduction in sulfate to sulfide, a key process in anaerobic respiration by SRB. The sulfide ions (S^2−^) produced can directly react with metal ions, forming metal sulfide precipitates and accelerating localized corrosion.

In SRB enrichment medium, SO_4_^2−^ is used as a terminal electron acceptor [15]. The general reduction in sulfate by SRB proceeds as follows:

SO_4_^2−^ + 8e^−^ + 10H^+^ → H_2_S + 4H_2_O (1)

On the cathodic sites of the metal surface, water reduction or hydrogen evolution may occur:

2H +2e^−^ → H_2_ (2)

Hydrogen gas or adsorbed hydrogen atoms (H) can serve as an electron donor for SRBs in the MET mechanism. The presence of SRB accelerates hydrogen uptake:

SO_4_^2−^ + 4H_2_ → HS^−^ + OH^−^ + 3H_2_O (3)

Dissolution of Al occurs in the anodic region:

Al → Al^3+^ + 3e^−^ (4)

The produced Al^3+^ ions may react with bisulfide ions (HS^−^), forming aluminum sulfide (Al_2_S_3_):

2Al^3+^ + 3HS^−^ + 3OH^−^ → Al_2_S_3_ + 3H_2_O (5)

Aluminum sulfide is unstable in water and hydrolyzes to form aluminum hydroxide and hydrogen sulfide gas:

Al_2_S_3_+ 6H_2_O → 2Al(OH)_3_ + 3H_2_S (6)

The overall electrochemical process of MIC on AA7075 in the presence of SRB can be represented as follows:

2Al + 3SO_4_^2−^ +6H_2_O → 2Al(OH)_3_ + 3HS^−^ + 3OH^−^ (7)

This study investigates the influence of SRB isolated from Lake Salda on the corrosion behavior of aerospace-grade aluminum alloys. By utilizing a comprehensive approach that includes electrochemical, microbiological, and molecular analyses, this research aims to achieve two main objectives: first, to enhance our understanding of how stromatolites in extreme environments like Lake Salda can provide valuable insights into past microbial life; and second, to clarify the corrosion mechanisms that may occur under such harsh conditions. Given Lake Salda’s unique geochemical and mineralogical characteristics, the findings are expected to provide important insights into microbial–metal interactions, biomineralization processes, and material degradation that are relevant to extreme terrestrial environments. These results will contribute to advancements in understanding the impact of microorganisms on structural materials in challenging conditions, which is essential for both fundamental scientific research and practical applications in material durability.

## 2. Materials and Methods

### 2.1. Sampling

**Figure 1:**
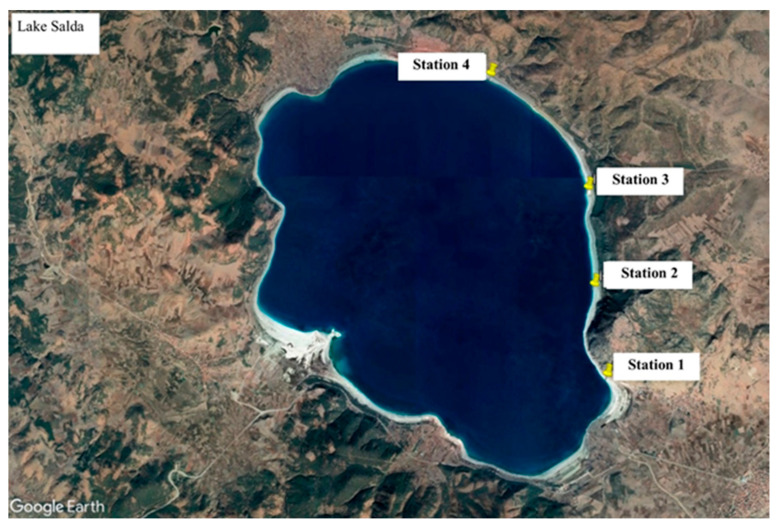
Sampling stations in Lake Salda.

Before sampling, the physical structure of Lake Salda was examined, some surveys were conducted along the shoreline, and the most suitable sampling points were selected. Sampling was not conducted in areas open to the public. Stromatolite, water, and biofilm samples were collected from the stations (Figure 1).

**Figure 2:**
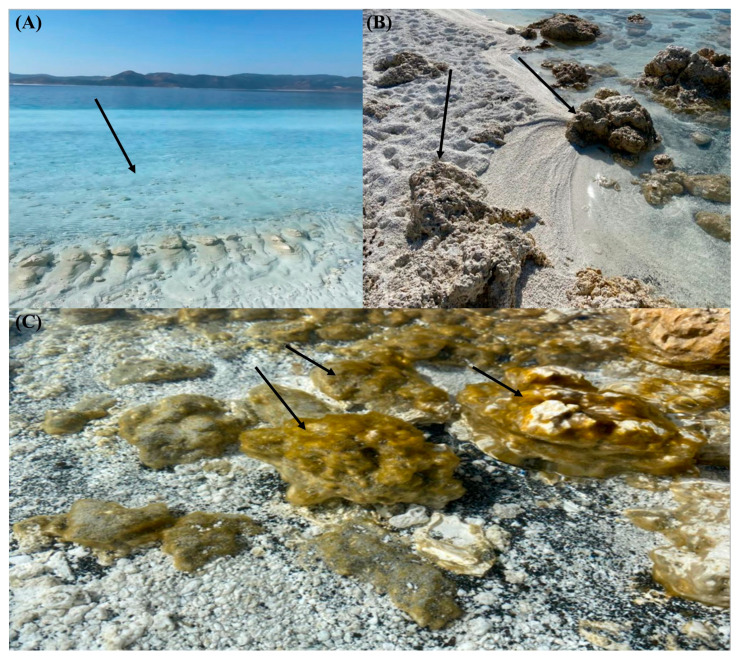
Sampling locations of Lake Salda (**A**) water, (**B**) stromatolites, (**C**) biofilm samples.

Water samples were taken from the surface of Lake Salda (approximately 5–10 m) under aseptic conditions (Figure 2A). The water samples were taken into sterile 1 L polypropylene bottles. All the samples were stored in the dark and used fresh within 6–8 h. Stromatolite samples were put in sterile Ziplock bags and transferred to the analysis laboratory immediately (Figure 2B). Biofilm samples were collected from the stromatolite’s surfaces using sterile swabs and then transferred into sterile Falcon tubes (Figure 2C).

### 2.2. Physicochemical Analyses of Lake Water

The physicochemical parameters of Lake Salda were systematically assessed to provide a comprehensive characterization of the water quality and environmental conditions. These parameters included dissolved oxygen (DO), total dissolved substances (TDS), total suspended solids (TSS), temperature, electrical conductivity (EC), and pH. Dissolved oxygen levels were measured using the classical Winkler titration method, a widely accepted and accurate technique for quantifying DO in aquatic environments [16]. TDS, EC, temperature, and pH were determined using a calibrated multiparameter portable device (AP-700, AQUAREAD). Water samples were filtered through pre-dried GF/F filter papers, which were subsequently dried at 105 °C and weighed to calculate TSS [16].

### 2.3. SRB Enrichment Culture and Cell Count

One liter of water samples taken from different depths were put into a sterile stomacher bag containing 20 mL of sterile Lake Salda water after passing through a sterile nylon membrane filter (50 mm in diameter and 0.22 μm pore diameter, Sartorius) and stored in the stomacher (IUL Instruments) for 2 min. Biofilm biomass collected from the stromatolite’s surface was deposited into a sterile tube containing 20 mL of Lake Salda water. After the homogenization, the dilution series were prepared starting from 10^−1^ to 10^−10^ for lake water and biofilm separately. Postgate B (PB) medium was used for the SRB enrichment culture. The medium’s pH was adjusted to 7.0 by using a NaOH solution. The medium was sterilized in an autoclave for 20 min at 121 °C. After autoclaving, the medium was sparged with filter-sterilized N_2_ to deoxygenate for at least one hour. All tests were carried out in anaerobic conditions. SRB were determined by the most probable number (MPN) technique. The prepared dilutions (1 mL) were inoculated into tubes containing 9 mL Postgate B medium. Also, sterile liquid paraffin was added in tubes to maintain anaerobic conditions. The tubes were incubated for 3 months at 37 °C. The black FeS precipitation in the tubes indicated the growth of SRB [17].

### 2.4. Microscopic Examination of Stromatolites

Stromatolite samples were immersed in a 10% hydrogen peroxide (H_2_O_2_) solution for 24 h to effectively break down organic matter and facilitate the separation of mineral components. Following this oxidation treatment, the samples were carefully rinsed and washed over a 0.063 mm sieve to remove any fine particulate matter and residual impurities. The residues were air-dried under controlled laboratory conditions to ensure complete moisture removal without altering the sample composition. Each size fraction was subsequently subjected to microscopic examination using optical microscopy techniques to characterize the morphology, texture, and any fossilized structures present within the stromatolite material.

### 2.5. Next Generation Sequencing (NGS)

One water sample was collected from Station 3 in October 2022. This sample was divided into two subsamples for enrichment purposes, and both subsamples were subjected to NGS. Bacterial DNA was isolated from the SRB enrichment culture obtained from the lake water using Qiagen DNeasy Blood and Tissue Isolation Kit (Tübingen, Germany). Subsequently, DNA extracts were visualized with agarose gel electrophoresis, and DNA density measurements were made with Nanodrop and Qubit devices. For bacterial 16S-rRNA genes, the V3–V4 region described by Kramar et al. (2019) [18] was amplified by PCR, which often captures a broad range of sulfur-metabolizing groups. In total, 16S rDNA library preparation processes with PCR products were performed, following the protocols described by Caporaso et al. (2012) [19] and Klindworth et al. (2013) [20]. Genetic libraries were prepared according to the 16S Metagenomic Sequencing Library Preparation Manual (Illumina, Cambridge, UK) for the V3 and V4 regions of the 16S gene. Sequencing was undertaken on the Illumina Nextseq platform with 150 bp PE sequencing, aiming for 300,000 reads per sample. The raw metagenomics data from the analyses were uploaded to GenBank. The data is available from the authors upon request. 

The quality of the raw read data obtained after Illumina sequencing was checked using FASTQC (Bioinformatics, Babraham, UK, 2011). CUTADAPT version 1.3 [21] was used to remove sequences that did not contain PCR primers and sequences shorter than 50 base pairs. The quality of the sequences was filtered based on a minimum Phred score and labeled in Qiime [22]. The resulting FASTA file was processed using the VSEARCH bioinformatics tool [23]. The sequences were subjected to dereplication using USEARCH [24], where all high-quality and cleaned sequences were consolidated into unique read sequences and clustered into OTUs. For taxonomic and phylogenetic analyses, assignments were made to the OTU matrix to determine which bacterial groups were present in the sample.

### 2.6. Coupons and Test Condition

AA7075 coupons with 25 × 25 × 10 mm dimensions were prepared. Coupons were supplied commercially, and their chemical composition is given in Table 1. Only the top surface was exposed to the culture media. All other surfaces were coated with silicon. Before each test, coupons were progressively abraded with 180, 400, 600, 800, and 1200 grit abrasive papers, then cleaned with 100% isopropanol and dried under UV light for at least 30 min. Afterwards, four coupons were placed in each of the 125 mL anaerobic vials containing 100 mL PB medium. Each vial was inoculated by adding 1 mL of 2-day-old seed culture isolated from Lake Salda. The vials were incubated at 37 °C for 7 days.

### 2.7. Enumeration of Sessile Cells

PB media were used to enumerate sessile cells using the MPN method. The coupons were gently rinsed with a pH 7.4. Phosphate-Buffered Saline (PBS) solution to remove planktonic cells. The biofilm on each coupon were removed with a sterilized brush-like applicator, and the biofilm constituents were vortexed for 1 min in 10 mL PBS solution. Then, the bacterial suspensions were serially diluted 10^7^ times to enumerate cells. MPN tubes were incubated at 37 °C. The MPN assay were repeated in triplicates.

### 2.8. Electrochemical Analyses

For electrochemical tests, a hole (2.5 mm diameter) was drilled on the edge of the coupons. Before each test, coupons were abraded with 180 up to 1200 grit abrasive papers progressively, then cleaned with 100% isopropanol, and dried under UV light for at least 30 min. The top of the coupon surface of 1 cm^2^ were exposed to the culture medium. All other surfaces were coated with hot silicon. Electrochemical tests were performed with a potentiostat (Gamry, Warminster, PA, USA) by measuring open circuit potential (OCP) and potentiodynamic polarization. All tests were carried out in 450 mL glass cells containing 300 mL PB medium and 3 mL seed culture isolated from Lake Salda. For the counter electrode, a platinum plate (10 mm × 10 mm × 1 mm) and for the reference electrode, a saturated calomel electrode (SCE) were used. AA7075 coupons (1 cm^2^ exposed surface) were used as working electrodes. Polarization curves were obtained with a scan rate of 1 mV/s between −250 mV and +250 mV vs. OCP. Corrosion current (*I*_corr_) values of the curves were determined from polarization curves.

### 2.9. Weight Loss Test

The biofilm and corrosion products were removed from the coupon surfaces according to ASTM G1–11 before weighing [25]. The weight losses of coupons and their corrosion rates were calculated. Corrosion rates were calculated using the following equation,

Corrosion rate (mdd) = (K × W)/(A × t × d)(8)

K = 3.45 × 10^6^
W = mass loss (g)
A = surface area (cm^2^)
t = time (h)
d = density (g/cm^3^)

### 2.10. FTIR (Fourier Transform Infrared) Analysis

The molecular structure and functional groups were identified using Fourier-Transform Infrared Spectroscopy with the Perkin Elmer FTIR 1600 spectrometer (Perkin Elmer Spectroscopy, Springfield, IL, USA) equipped with an Attenuated Total Reflectance (ATR) accessory. For this analysis, a 100-microliter sample was subjected to an infrared scan covering the range of 400 to 4000 cm^−1^, with 16 scans at a resolution of 4 cm^−1^ [26].

### 2.11. Surface Analyses

After removing biofilm and corrosion products, pit morphologies on the coupon surfaces were analyzed using scanning electron microscopy (SEM). Before SEM analysis, all the coupons with biofilm were fixed with a 4% (*w*/*w*) glutaraldehyde solution for 2 h to preserve cellular structures and prevent any morphological alterations during subsequent sample preparation, then dehydrated in a graded series (25%, 50%, 75%, and 100% by volume) of isopropanol. The dried coupons were coated with palladium to provide conductivity using a sputter coater and imaged with a high-resolution field emission scanning electron microscope (Model FEI Quanta 450 FEG).

## 3. Results

### 3.1. Physicochemical Analyses of Lake Water

The physicochemical characteristics of the water samples are summarized in Table 2. Across all sampling stations, the pH values remained relatively consistent, averaging approximately 10.0. Electrical conductivity (EC) showed no significant variation among the stations, with the highest recorded value being 1.36 mS/cm at station two. Water temperature ranged from approximately 16.3 °C to 20.4 °C. Dissolved oxygen (DO) concentrations peaked at 8.74 mg/L at station one, while the lowest value of 7.34 mg/L was observed at station two. Total dissolved solids (TDS) reached a maximum of 1723 mg/L at station two compared to other sampling sites. The highest concentration of total suspended solids (TSS) was recorded as 15 ppm at station four.

### 3.2. Microscopic Examination of Stromatolites

**Figure 3:**
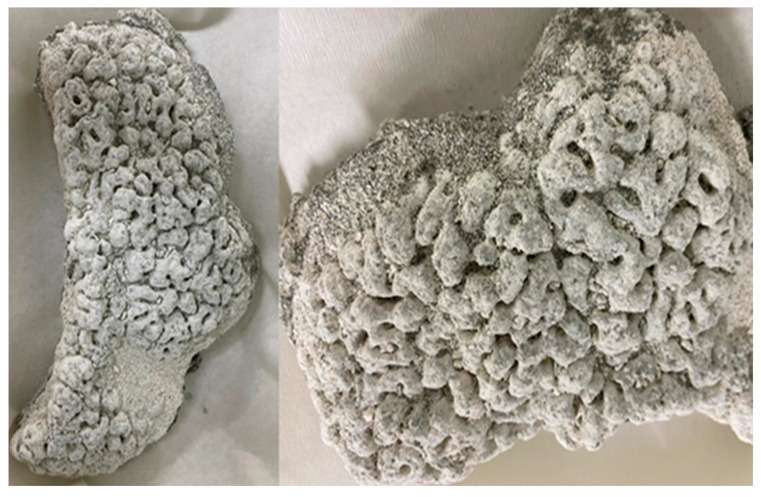
Stromatolite samples from Lake Salda.

**Figure 4:**
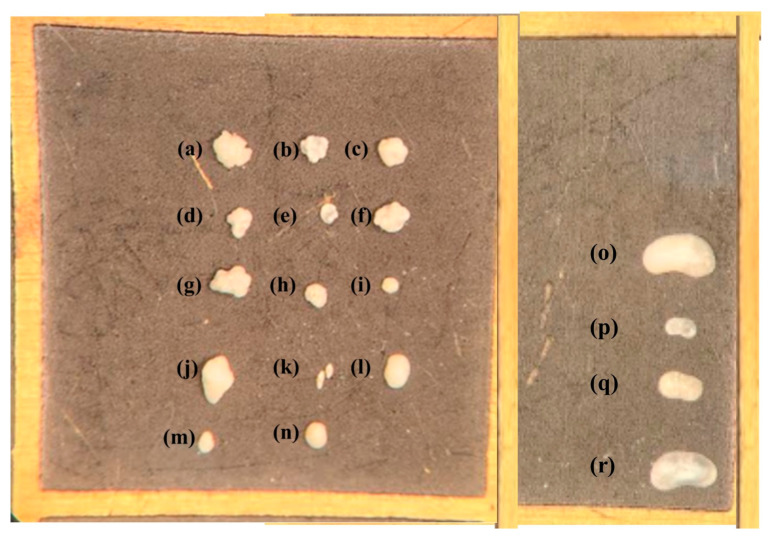
Benthic microorganisms isolated from stromatolite samples (**a–h**) *Ammonia* sp., (**i**) *Ammonia* tepida, (**j**) *Protoglobobulumina* sp. (**k**) *Fursenikonia acuta*, (**l**) *Gavelinopsis* sp., (**m**) *Rosalina* sp., (**n**) *Conorbella* sp., (**o**) *Candona neglecta*, (**p**) *Ilyocypcis bicornis*, (**q**) *Paralimnocythere bicornis*, (**r**) *Candona angulata*.

Salda stromatolites taken from the lake were cauliflower-shaped and consisted of small domes (Figure 3). Most of the samples were covered with a thick biofilm layer. During microscopic examinations, the presence of transported and recrystallized benthic foraminifera among the biogenic components were identified. Six different genera of benthic foraminifera were identified (Figure 4). These are *Ammonia* sp., *Conorbella* sp., *Fursenkoina* sp., *Gavelinopsis* sp., *Protoglobulimina* sp., and *Rosalina* sp. *Ammonia* sp. is the most relatively abundant species among the identified forms. The genera and species of these species are euryhaline types capable of living in marine environments with varying salinity levels (0–50 m depth). Ostracods ranging in size from 200 to 800 μm were also observed (Figure 4n–p). In stromatolite samples, four different ostracod species were identified: *Ilyocypris biplicata*, *Candona angulata*, *Candona neglecta*, and *Paralimnocythere bicornis*.

### 3.3. SRB Enrichment Culture and Cell Count in Biofilm and Lake Water

**Figure 5:**
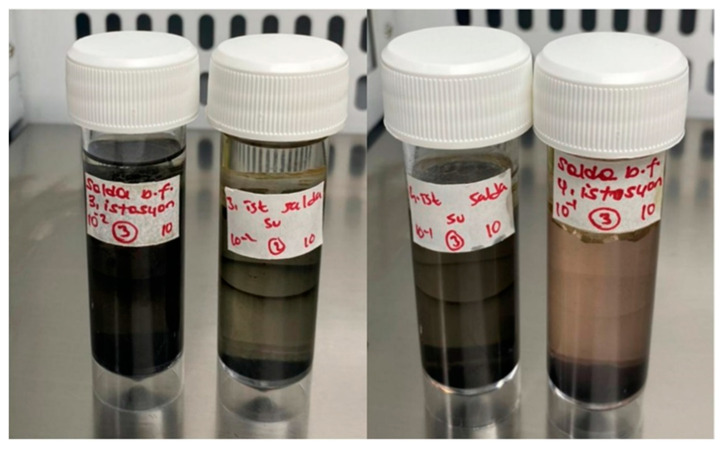
SRB detection in biofilm formed on the stromatolite’s surface and Lake Salda water.

**Figure 6:**
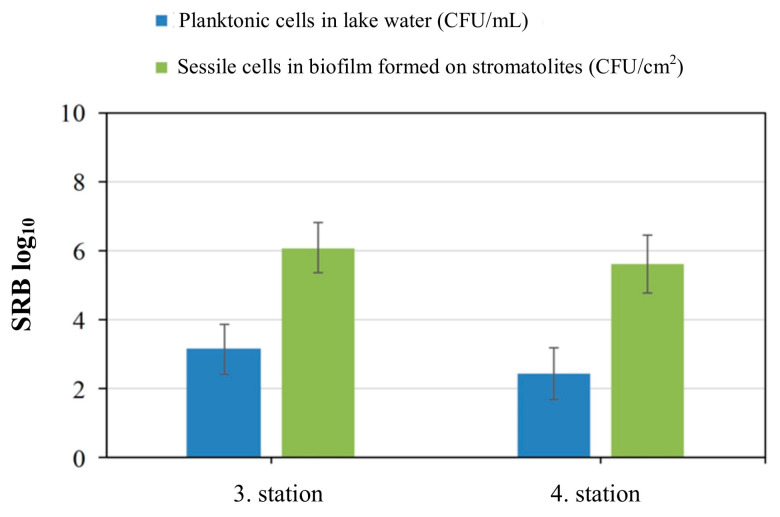
Planktonic and sessile cells in lake water and biofilm formed on stromatolites.

SRB were isolated from biofilm samples formed on the stromatolite’s surfaces and in the Lake Salda water. All samples were inoculated into the SRB enrichment culture. After the incubation time, the tubes turned black in color, and the best growth was observed from the third and fourth stations (Figure 5). The total bacteria cell counts are given in Figure 6. The total cell count in the lake water samples obtained from the third station was found to be 5180 cfu/mL, while at the fourth station, it was 3877 cfu/mL. The total cell count in the biofilm samples was found to be 4.8 × 10^5^ cfu/cm^2^ in the third station and 4.4 × 10^5^ cfu/cm^2^ in the fourth station. Also, it was found that the total cell count in the biofilm was significantly higher than in the water (*p* < 0.05), and a significant positive correlation was detected between the bacterial counts (r = 0.846, *p* < 0.01). The experiment was repeated twice for reproducibility.

**Figure 7:**
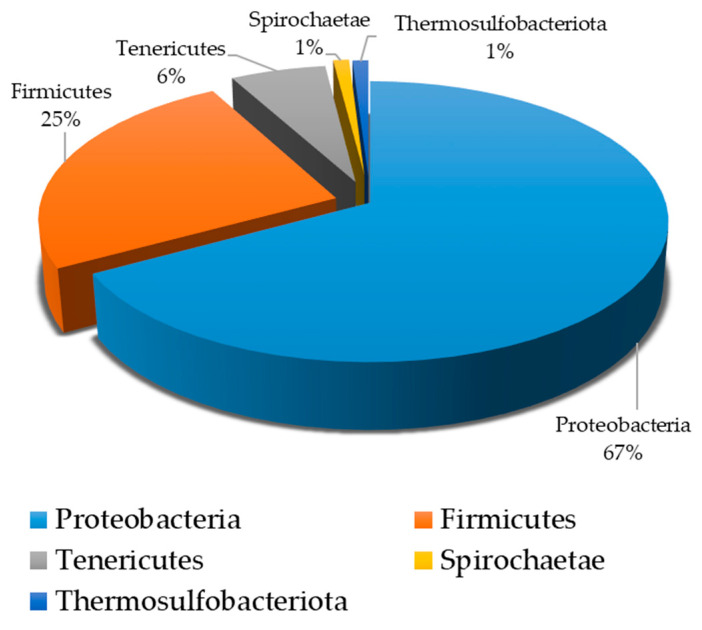
Percentage of bacterial phyla in water samples obtained from Lake Salda.

The presence of SRB in Lake Salda’s water was also confirmed using a molecular approach. The NGS analysis results showed that the SRB coexisted with other bacteria in the water samples taken from Lake Salda (Table 3). The dominant phyla in this study included Proteobacteria (67%), Firmicutes (25%), Tenericutes (˂6%), Spirochaetae (˂1%), and Thermosulfobacteriota (˂1%) (Figure 7). In Lake Salda’s water, the first time, *Desulfofustis limnaeus*, a novel member of the Thermosulfobacteriota was identified. The alpha diversity, counted as number of species, was 60. The number of raw reads, reads left after post quality filtering, and number and proportion of reads used in final classification are given in Table 4.

### 3.4. Biofilm Observations, Cell Count and Corrosion Analyses

**Figure 8:**
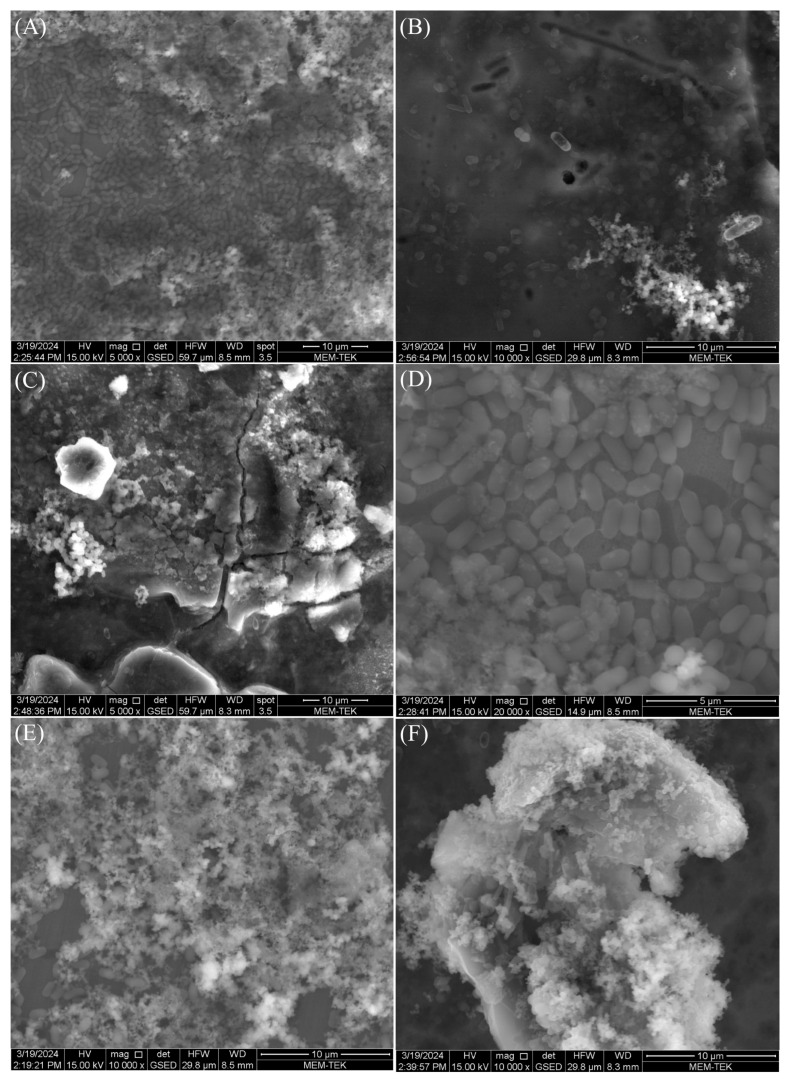
SEM images of SRB biofilm (**A**–**E**) and corrosion product (**F**) on AA7075 coupons after the 30-day incubation.

**Figure 9:**
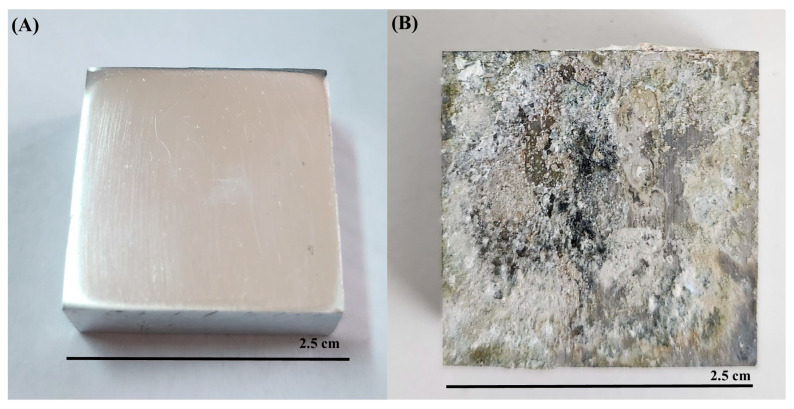
Visual images of AA7075 alloy (**A**) fresh coupon, (**B**) after 30-day incubation.

**Figure 10:**
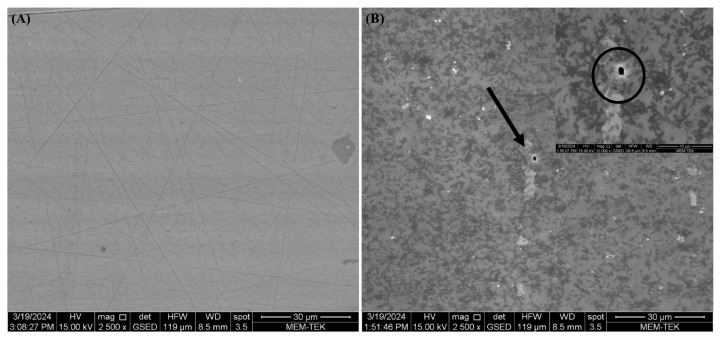
SEM images of pits on AA7075 coupons: (**A**) fresh coupon (**B**) after 30-day incubation.

All microorganisms identified in lake water and their metabolic activities can influence the corrosion mechanisms on AA7075 through the formation of biofilms and anodic and cathodic reactions. After a 30-day incubation time, SEM images showed that a heterogeneous biofilm was formed on the coupon’s surface (Figure 8). Bacterial cells were embedded in the corrosion product layer (Figure 8B). EPS in biofilm composed of polysaccharides, proteins, and thiol groups play a crucial role in accelerating the corrosion of the AA7075 alloy. The metabolic activity of SRB creates a microenvironment that disrupts the protective oxide layer through electron transfer, facilitating sulfate reduction to sulfide ions which react with metal ions to form corrosion products. Also, the EPS matrix traps aggressive chloride ions and moisture, promoting localized corrosion. Thiol groups within EPS form strong complexes with metal ions, weakening the passive film and enhancing metal dissolution. Thus, the chemical composition of biofilm EPS directly influences the corrosion mechanisms observed. The sessile cells were very abundant, as shown in Figure 8E. Also, the surfaces of the coupons exposed to the culture medium were covered with irregular corrosion products (Figure 8F). For raw SEM images see the Supplementary Figure S1. The sessile cell counts on the test coupons were found to be 10^6^ cells/cm^2^ after a 30-day incubation. Visual images of the test coupons also showed that the AA7075 alloy underwent corrosion in the presence of biofilm after the 30-day incubation (Figure 9B). The corrosion products covered the entire surface of the alloy. Also, localized corrosion areas were observed on the surface of the coupons. However, it was seen that the surface morphology of AA7075 coupons was completely changed by biofilm. These results indicated that a mixed species consortium isolated from Lake Salda accelerated aluminum dissolution. In Figure 10A, the polishing lines are seen on a fresh coupon. After removing the biofilm and corrosion products, some damage and pits are clearly observed on the AA7075 coupon surface (Figure 10B).

**Figure 11:**
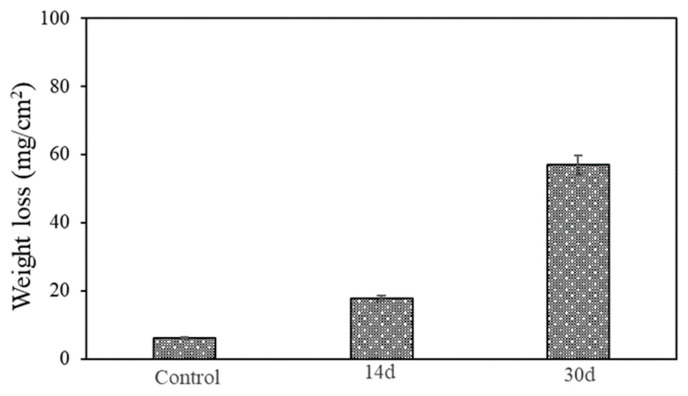
Weight losses of coupons after 14-day and 30-day incubation in vials.

The weight loss data of the AA7075 alloy are shown in Figure 11. Each weight loss data point represents the average from the replicate coupons. The weight loss for the control coupon was the lowest (6.1 ± 0.1 mg/cm^2^) after the 30-day incubation. The weight losses (mg/cm^2^) were 17.8 ± 0.25, 57.0 ± 0.20 and the corrosion rates were calculated as 1.71 mm/y and 2.56 mm/y for after 14-day and 30-day incubation, respectively. Note that the removal of the corrosion products on reactive metals such as Al is quite difficult because of the oxide layer tightly bonded to the surface.

**Figure 12:**
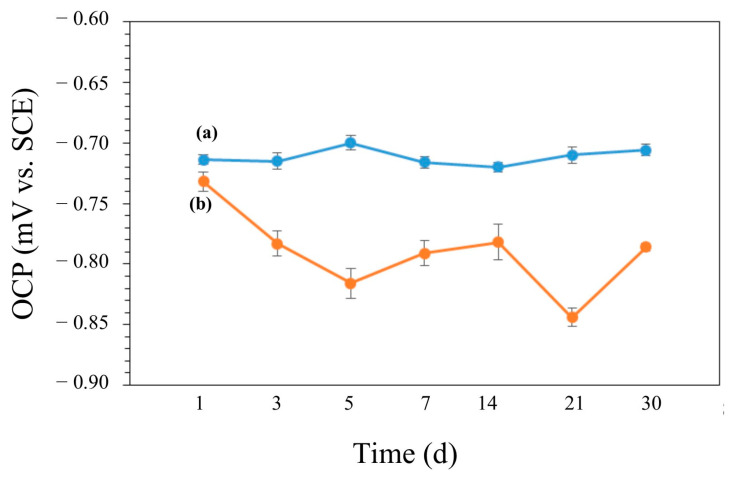
OCP values for (**a**) control and (**b**) biotic coupons.

**Figure 13:**
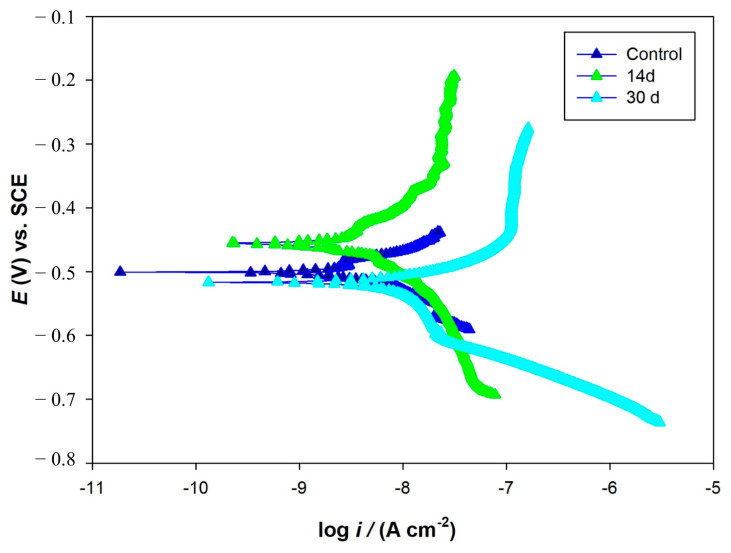
Potentiodynamic polarization curves for control and biotic coupons.

The variations in OCP vs. time during the 30-day incubation in the media with and without bacteria are shown in Figure 12. The OCP value of the coupon was around −0.714 V in the abiotic medium and it did not show significant changes during the experiment (Figure 12a). In the biotic medium, the OCP value of the coupon shifted into the negative during the 30 days of incubation (Figure 12b). More negative values in *E*_ocp_ were also observed for the biotic coupons, also indicating increased tendencies for electron loss by AA7075. PDP scans are shown in Figure 13. The electrochemical parameters (*E*_corr_, *I*_corr_, *β*_α_, *β*_c_) are given in Table 5. The control coupon showed lower corrosion current density (*I*_corr_) compared to the biotic coupons. The *I*_corr_ values of the AA7075 coupons were found to be 9.2 and 76.7 nA/cm^2^ after 14 and 30 days of incubation, respectively. For PDP data see the Supplementary Table S1.

**Figure 14:**
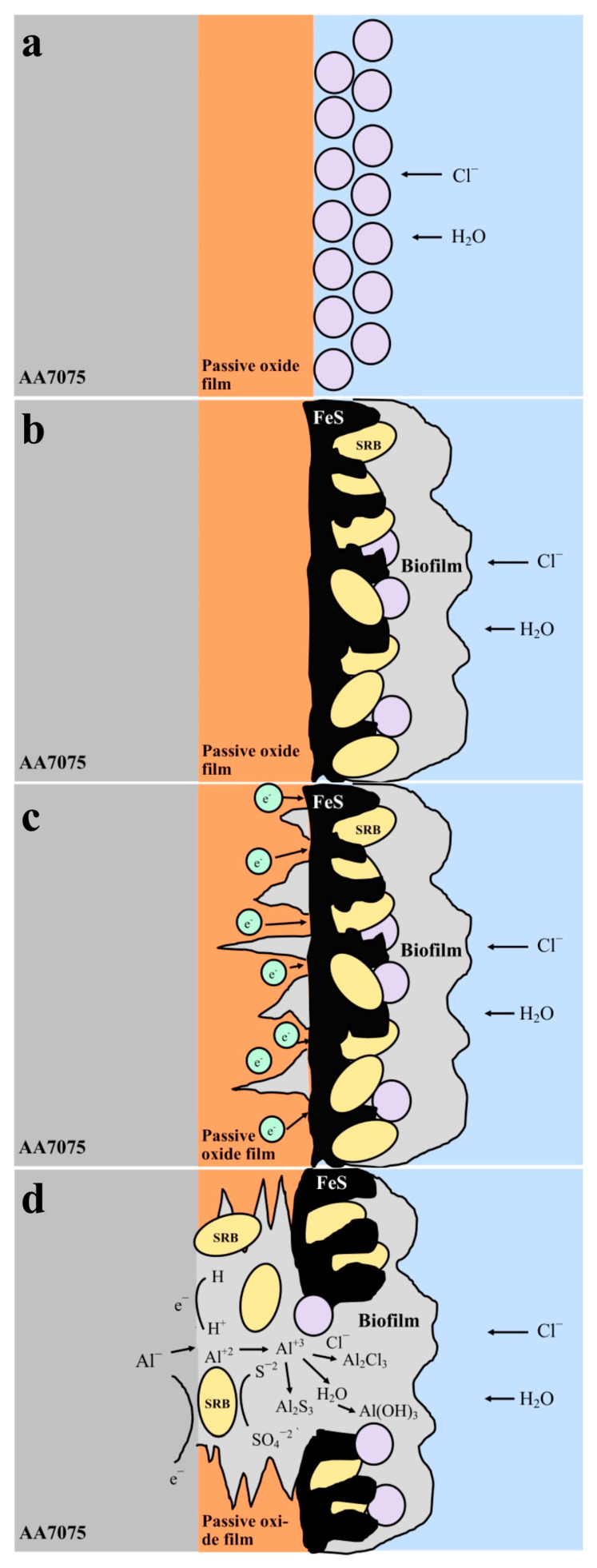
Generalized illustration of the corrosion mechanism for the 7075 aluminum (**a**) in sterile solution, (**b**–**d**) in SRB-enriched media.

In Figure 14, the schematic illustrates the MIC process of the AA7075 aluminum alloy in the presence of SRB. Initially, the alloy surface is protected by a passive oxide film that inhibits the ingress of chloride ions and water (Figure 14a). Over time, SRB colonize the surface, forming a biofilm and producing corrosion byproducts such as FeS (Figure 14b). The biofilm induces localized degradation of the passive layer, thereby facilitating the penetration of aggressive ions (Cl^−^, S^2−^, H^+^) to the underlying metal substrate (Figure 14c). In the final stage, extensive deterioration of the passive film occurs, and the metabolic activity of SRB promotes the formation of corrosion products including AlCl_3_, Al(OH)_3_, and Al_2_S_3_ through electrochemical reactions (Figure 14d).

### 3.5. FTIR Analyses

**Figure 15:**
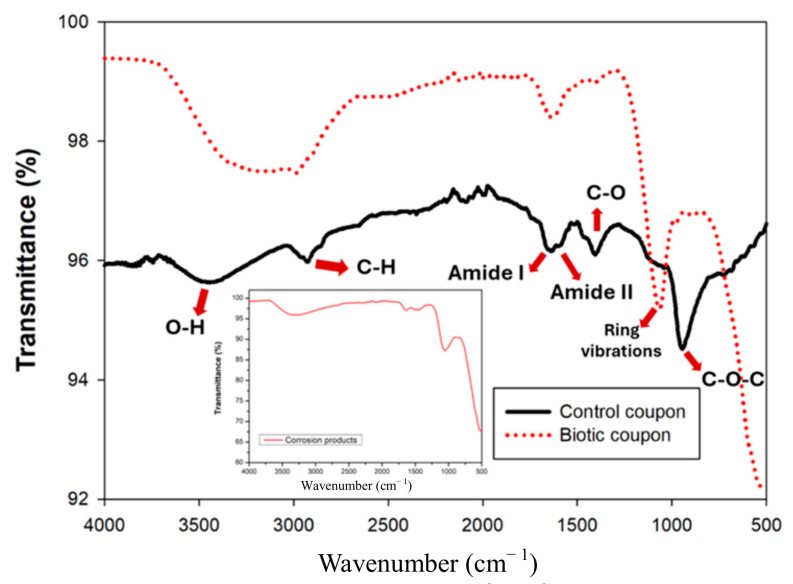
FTIR results of control, biotic coupons and corrosion product on AA7075 alloy.

The FTIR spectra revealed clear differences between the control coupon and biotic coupons (Figure 15). The control coupon exhibited distinctive absorption features. Notably, a broad O–H stretching band was observed near 3300 cm^−1^, while an aliphatic C–H stretch, characteristic of fatty acids, appeared at 2947 cm^−1^. The amide I and II bands, indicative of protein components, appeared at 1658 cm^−1^ and 1566 cm^−1^, respectively. Additionally, a band around 1419 cm^−1^ corresponded to C–O bending vibrations of carboxylate ions, and a prominent peak near 980 cm^−1^ was attributed to C–O–C linkages in polysaccharides [27]. These spectral features reflect the molecular composition of the culture medium adsorbed onto the aluminum coupon surface. In contrast, the biotic coupons showed a distinct shift within the polysaccharide-associated region. The absorption band at 980 cm^−1^ was no longer evident, whereas a prominent new band emerged at 1075 cm^−1^, attributable to the ring vibrations characteristic of cyclic polysaccharides (Figure 15). This spectral modification suggests the synthesis of a bacterial biofilm matrix, as EPS are known to be rich in polysaccharides with cyclic structural features [28]. Consequently, the appearance of the 1075 cm^−1^ band serves as molecular evidence for biofilm maturation and EPS formation on the AA7075 aluminum surface under SRB-enriched conditions. The findings collectively reveal that the transition from PB medium to SRB biofilm is marked by distinctive chemical signatures, most notably the appearance of the 1075 cm^−1^ cyclic polysaccharide vibration. This spectral feature correlates directly with biofilm development and matrix synthesis, which subsequently indicate the MIC of AA7075 under extreme environmental conditions. For raw FTIR data see the Supplementary Table S2.

## 4. Discussion

The physicochemical properties of Lake Salda’s water play a critical role in shaping microbial communities and understanding corrosion processes. In this study, the pH values were found to be similar at all stations. Balcı et al. (2018) reported that the pH of lake water ranges from 8.5 to 9.2, and the temperature values were around 17.8 °C to 35.4 °C [6]. This variability suggests alterations in the lake’s chemical composition. Kazancı et al. (2004) [29] suggested that this change occurs due to the overturning of the water column in April and October. Moreover, evaporation, the use of groundwater for irrigation purposes, and karstic aquifers around the lake have been identified as causes of the annual water level fluctuation [29].

The presence of eukaryotic organisms, such as foraminifera and ostracods, within stromatolitic structures can significantly influence the composition, spatial organization, and nutrient cycling of microbial communities. Although these organisms are not microorganisms themselves, their interactions with microbial populations can shape the overall structure and function of the community [30,31,32]. Their physical forms and metabolic byproducts may either promote or inhibit microbial colonization and biofilm formation on mineral or metallic surfaces. Furthermore, identifying the macro and microorganisms present within an ecosystem provides important ecological context. This information reveals environmental conditions such as salinity, oxygen levels, and organic matter availability, all of which directly impact microbial metabolic pathways. Many of these pathways, including sulfate reduction and iron oxidation, are closely associated with MIC processes. The presence of specific taxa also influences key factors such as mineral precipitation, EPS production, and localized pH conditions. All of these factors play critical roles in initiating and progressing corrosion. In summary, observations made at the microscopic level, along with data on species composition, are not merely descriptive; they are essential for interpreting microbial dynamics and understanding corrosion processes.

The presence of SRB in Lake Salda may provide insight into how the first forms of life on Mars existed and how biogeochemical processes occurred in anaerobic conditions. Researchers reported that the presence of SRB and sulfate reduction evolved at least 3.7 Ga ago based on the isotopic data, even before the evolution of oxygenic photosynthesis and cyanobacteria [33,34]. Microbial sulfate reduction by SRB leads to sulfur isotopic fractionation, which typically results in a depletion of the isotope ^34^S in the produced sulfide. This isotopic signature, represented by δ^34^S values, serves as a strong geochemical indicator of ancient microbial activity and can be used to differentiate between biotic and abiotic sulfur cycling. Data collected from Lake Salda and similar environments may provide a baseline for interpreting potential biosignatures on Mars. Balcı et al. (2018) [6] reported that 97.3% and 2.7% of the prokaryotic population of Lake Salda belongs to the bacteria and archea domains, respectively [6]. They also pointed out that the fossil and present-day stromatolite formation may provide important clues about traces of early life preserved in the geological records. According to NASA’s Curiosity rover data, Mars may have been inhabited in the ancient past [35]. Several scientists have also hypothesized that many species, including SRB, may have colonized Mars [36]. The dominant phyla in this study included Proteobacteria (67%), Firmicutes (25%), Tenericutes (˂6%), Spirochaetae (˂1%), and Thermosulfobacteriota (˂1%) (Figure 7). Balcı et al. (2020) reported that the bacteria phyla in Lake Salda consist of Proteobacteria (55.2%) and Firmicutes (30.1%), Cyanobacteria (7.3%), Actinobacteria (1%), and Bacteroidetes (1%), and other phyla (less than 1%) [37]. *Desulfofustis limnaeus*, a novel member of the Thermosulfobacteriota was identified for the first time in Lake Salda’s water. However, its percentage in the total culture was found to be less than 1% (Figure 7). *Desulfofustis limnaeus* is a freshwater SRB that has been isolated from marsh soils. This bacterium carries out dissimilatory sulfate reduction using various organic compounds as electron donors, including lactate, malate, formate, fumarate, and succinate. It employs sulfate, sulfite, thiosulfate, and elemental sulfur as electron acceptors. Moreover, when other carbon sources such as yeast extract and acetate are present, hydrogen can also act as an electron donor. The metabolic activity of *D. limnaeus* leads to the production of H_2_S, which can chemically interact with metals and contribute to MIC [38].

The PDP results indicated that SRB biofilm notably accelerated the corrosion of the AA7075 alloy. Some researchers have also reported similar results for AA7075 alloy in SRB in the marine environment [39,40]. Vejar et al. (2016) [41] reported that in the presence of *Bacillus megaterium,* the corrosion current increased threefold in comparison with the control coupon after a 14 d incubation [41]. Also, Zhang et al. (2022) [42] found that the fungus *Aspergillus terreus* can survive with organic carbon starvation and accelerate AA7075 corrosion [42]. Some passivity regions were also found on PDP curves. This may be due to the accumulation of corrosion products during incubation time. Polarization curves of AA7075 for 14 d and 30 d showed a sharp increase in the anodic current density directly above corrosion potential (Figure 13). The formation of biofilm on AA7075’s surface may accelerate the damage of the surface passive film and then increase the localized corrosion rate. Pit images also supported these results (Figure 9B). The EPS matrix and H_2_S produced by SRB create a localized drop in pH at the interface between the metal and the biofilm. This acidic environment enhances the mobility and penetration of aggressive chloride ions, which can destabilize the passive oxide layer. As a result, this process promotes localized corrosion, particularly the initiation and growth of pits. Guan et al. (2020) [43] reported that the localized corrosion of Al alloys was increased by SRB [43]. Figure 14 shows how SRB biofilms disrupt the passive protection layer and accelerate the corrosion of the AA7075 alloy. The findings in this study also indicate that the aluminum surface becomes increasingly vulnerable to MIC caused by SRB over time. Especially during the log phase, the increased biological activity of SRB leads to the formation of biofilm and metabolic products that damage the oxide layer on the aluminum surface. This allows direct electron transfer to occur. Electrochemical tests confirm clear signs of corrosion at this stage.

## 5. Conclusions

This study demonstrates that SRB and other anaerobic microorganisms isolated from Lake Salda have a significant impact on the MIC behavior of AA7075 alloy, which is widely used in aerospace applications. While the microbial ecosystem in Lake Salda is complex and consists of multiple interacting species, this study specifically focused on SRB because of their known role in MIC and their potential relevance to survival under extreme conditions. Therefore, SRB were selected as a model group to explore MIC processes under anaerobic conditions relevant to extreme environments like Lake Salda. Microscopic and molecular analyses confirmed a high abundance of SRB in both the lake water and stromatolite-associated biofilms. The SRB consortium formed robust biofilms on AA7075 surfaces during the 30-day incubation period, leading to accelerated corrosion. Although aluminum and its alloys are known for their superior corrosion resistance due to the formation of a protective oxide layer, in the presence of an SRB consortium, AA7075 is not fully immune to MIC pitting attacks. PDP, FTIR, and SEM analyses confirmed that microbial activity significantly changed the surface morphology and increased the corrosion rates.

The detection of *Desulfofustis limnaeus* in the lake for the first time, alongside dominant microbial phyla such as Proteobacteria and Firmicutes, highlights the rich microbial diversity adapted to hypersaline and alkaline conditions. In conclusion, the findings point to the importance of considering microbial activity in the selection and testing of materials intended for use in extraterrestrial environments. Lake Salda is one of the best natural testbeds on Earth for planetary exploration and spacecraft materials. However, the environmental conditions in our experiments (e.g., temperature, nutrient availability, and atmospheric composition) differ significantly from those on Mars. As such, the findings of this study are not intended to directly replicate Martian corrosion processes but rather to offer analog insights under controlled conditions that support microbial activity.

The recent detection of greigite and vivianite in Martian samples collected by NASA’s Perseverance rover [9] enhances the relevance of Earth’s extreme environments, such as Lake Salda, as analog sites for astrobiological studies. These minerals are commonly associated with SRB in anoxic terrestrial environments, where microbial processes contribute to their formation. In Lake Salda, SRB-mediated interactions may similarly promote the development of phosphate and sulfide minerals within stromatolitic structures. Although the presence of greigite and vivianite on Mars does not constitute definitive evidence of past life, studying their formation in Earth analogs can provide valuable insights into possible biosignatures and guide future exploration strategies.

## Figures and Tables

**Table 1:** Elemental composition of AA7075 alloy.

Element	Fe	Si	Cu	Mn	Cr	Ti	Mg	Zn	%Al
Amount (wt%)	0.10	0.05	1.60	0.05	0.18	0.05	2.50	5.80	Balance

**Table 2:** Physicochemical parameters of Lake Salda water.

Parameters	Station 1	Station 2	Station 3	Station 4
pH	10.16	10.01	10.08	10.04
EC (mS/cm)	1.21	1.36	1.24	1.21
DO (mg/L)	8.74	7.34	7.98	8.26
TDS (ppm)	1715	1723	1610	1540
Temperature (°C)	16.3	17.8	18.9	20.4
TSS (mg/L)	2	2	4	15

**Table 3:** Some bacterial species isolated from Lake Salda (NGS results).

Species	Similarity
*Entomomonas asaccharolytica*	100
*Mycoplasma* sp. *(ex Biomphalaria glabrata)*	100
*Pseudomonas knackmussii*	100
*Pseudomonas oryziphila*	100
*Salmonella enterica*	100
*Pseudomonas mosselii*	100
*Escherichia coli*	100
*Halopseudomonas litoralis*	100
*Mycoplasmoides fastidiosum*	100
*Pseudomonas* sp. BT-42-2	100
*Lactiplantibacillus paraplantarum*	100
*Comamonas antarctica*	100
*Weissella hellenica*	100
*Intestinimonas butyriciproducens*	100
*Enterococcus faecium*	100
*Bacillus thuringiensis*	100
*Pseudomonas mediterranea*	100
*Pseudomonas* sp. Os17	100
*Halomonas* sp. MS1	100
*Candidatus Pseudomonas adelgestsugas*	100
*Kangiella sediminilitoris*	100
*Desulfofustis limnaeus*	100

**Table 4:** Read statistics (SG: Lake Salda).

Sample Name	Number of Raw Reads	Reads After Filtering	Number and Proportion of Reads Used in Final Classification
SG1	294.112	122.857	187.728

**Table 5:** Electrochemical parameters derived from the Tafel analysis of coupons in medium with and without bacteria.

Coupon	*E*_corr_ (mV) vs. SCE	*I*_corr_ (nA/cm^2^)	*B*_a_ mV/dec	*B*_b_ mV/dec
Control	−500	2.4	126	149
Biotic (14 d)	−456	9.2	100	834
Biotic (30 d)	−518	76.7	161	77

## Data Availability

The original contributions presented in this study are included in the article. Further inquiries can be directed to the corresponding author.

## Supplementary Materials

The following supporting information can be downloaded at: https://www.mdpi.com/article/10.3390/microorganisms13112555/s1, Figure S1: Raw SEM images; Table S1: PDP data; Table S2: Raw FTIR data.
