# Supplementary figures and images for: Microbiologically Influenced Corrosion of Aerospace-Grade Aluminum by SRB-Enriched Biofilms Isolated from the Mars Analog Lake Salda
Source: Microorganisms. 2025 Nov 8;13(11):2555. doi: 10.3390/microorganisms13112555 (PMC12654286; doi:10.3390/microorganisms13112555)

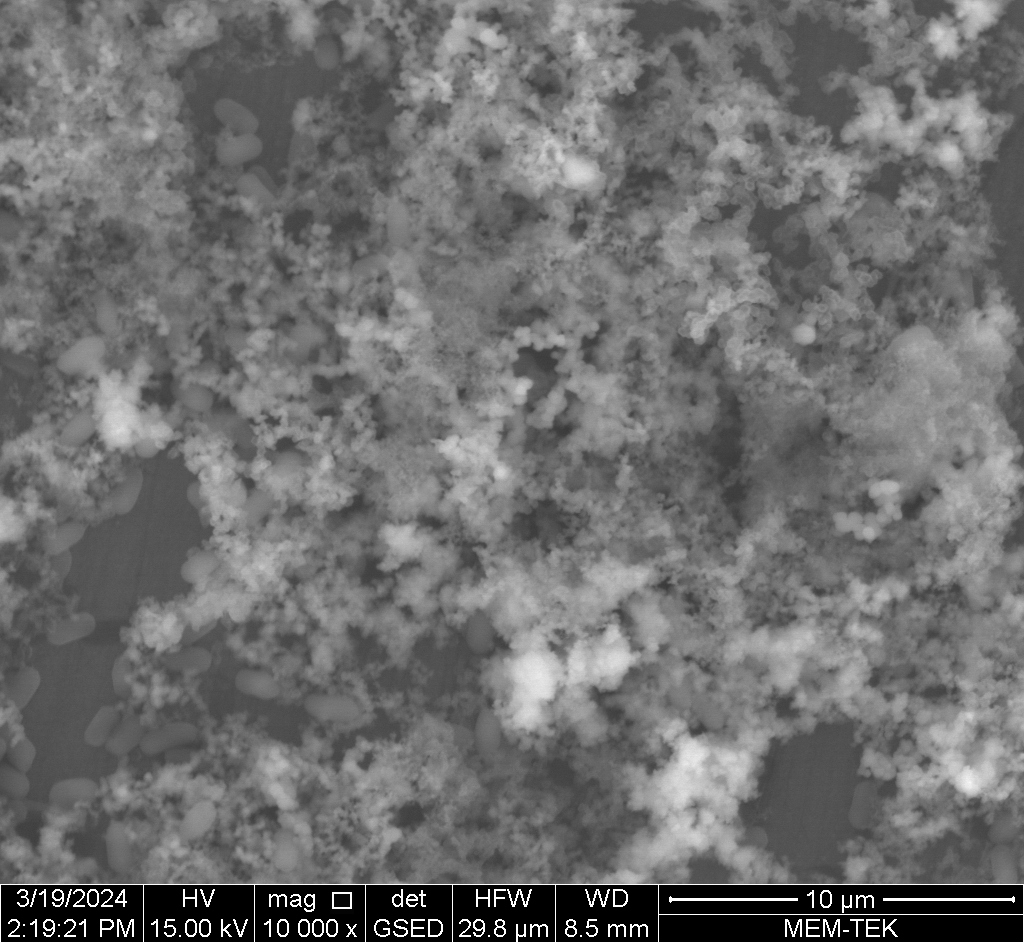

Supplement: Supplementary file 1 [file microorganisms-13-02555-s001.zip › Figure S1. 2024.03.19_SEM/3_1.tif]

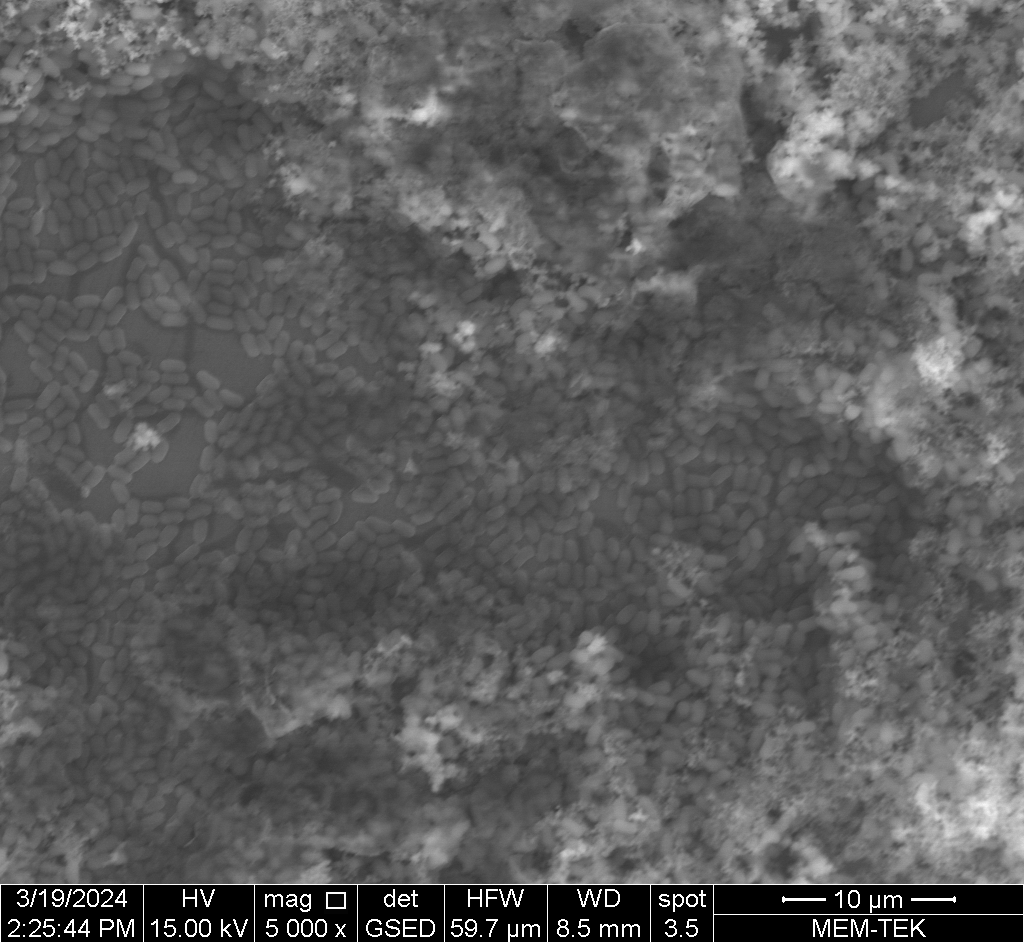

Supplement: Supplementary file 1 [file microorganisms-13-02555-s001.zip › Figure S1. 2024.03.19_SEM/3_2.tif]

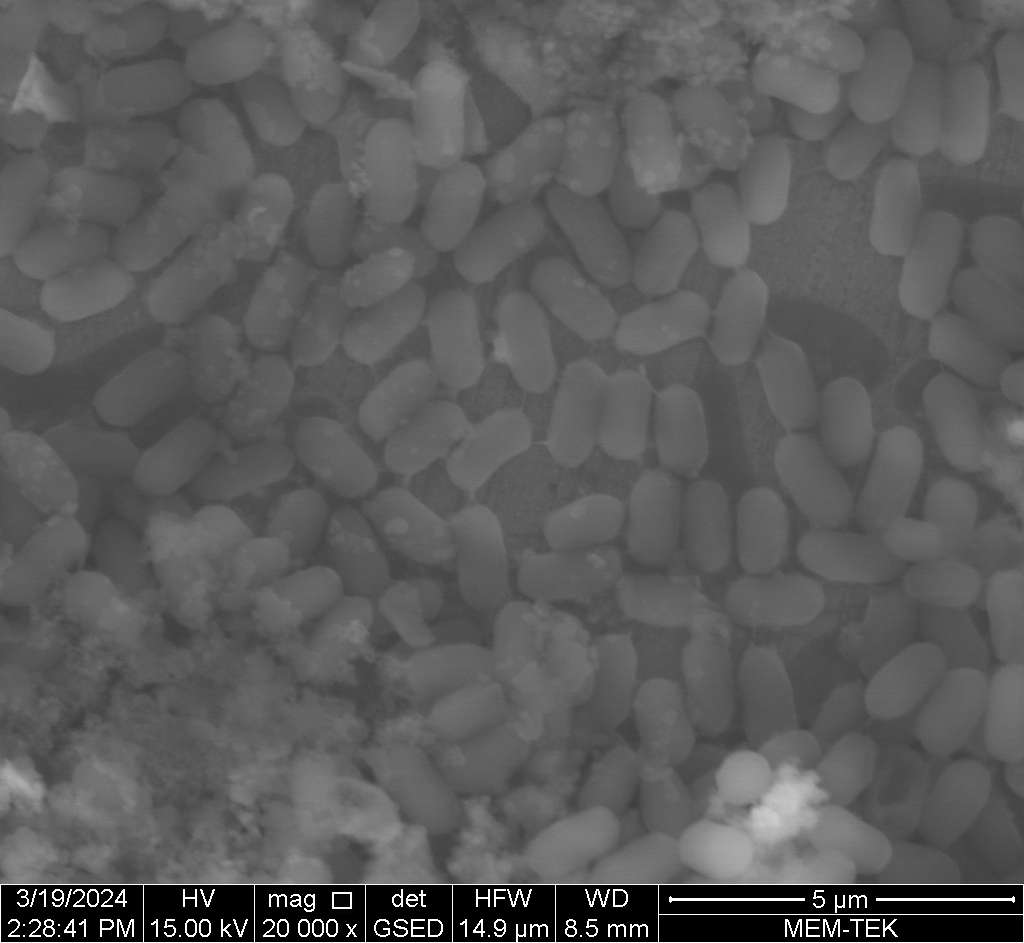

Supplement: Supplementary file 1 [file microorganisms-13-02555-s001.zip › Figure S1. 2024.03.19_SEM/3_3.tif]

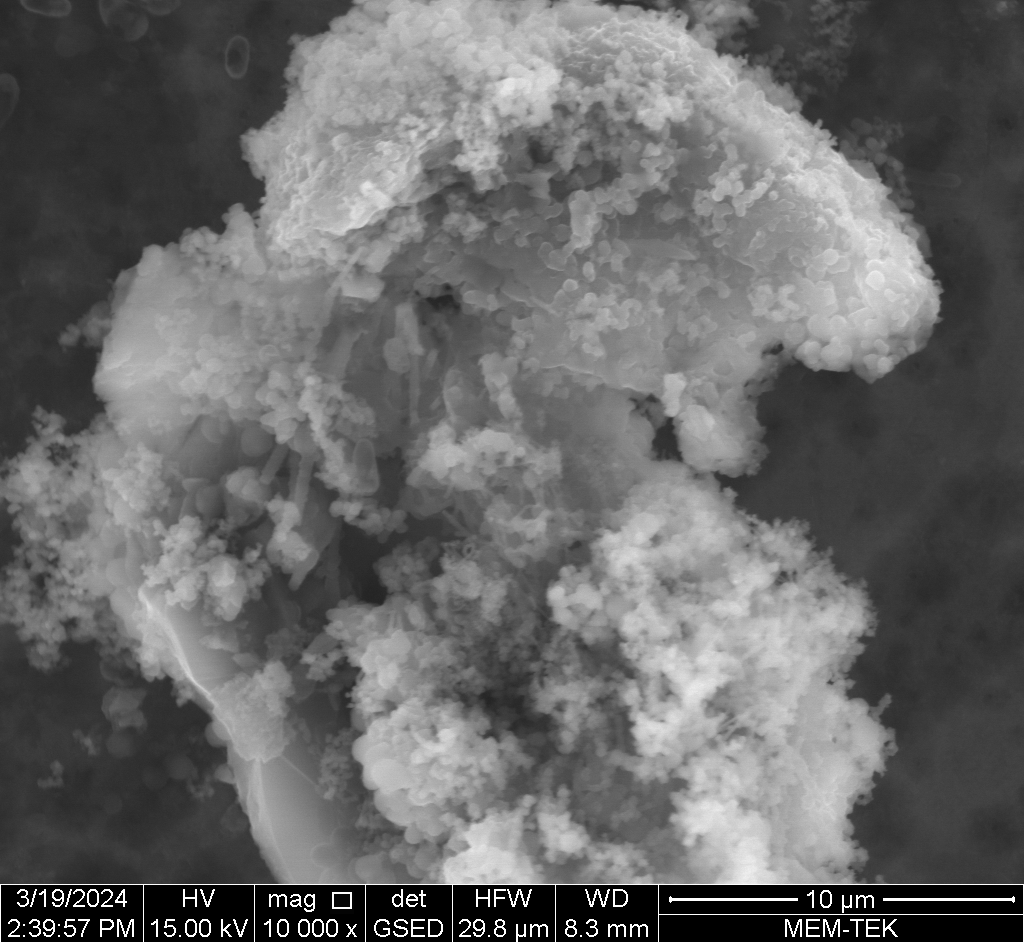

Supplement: Supplementary file 1 [file microorganisms-13-02555-s001.zip › Figure S1. 2024.03.19_SEM/3_4.tif]

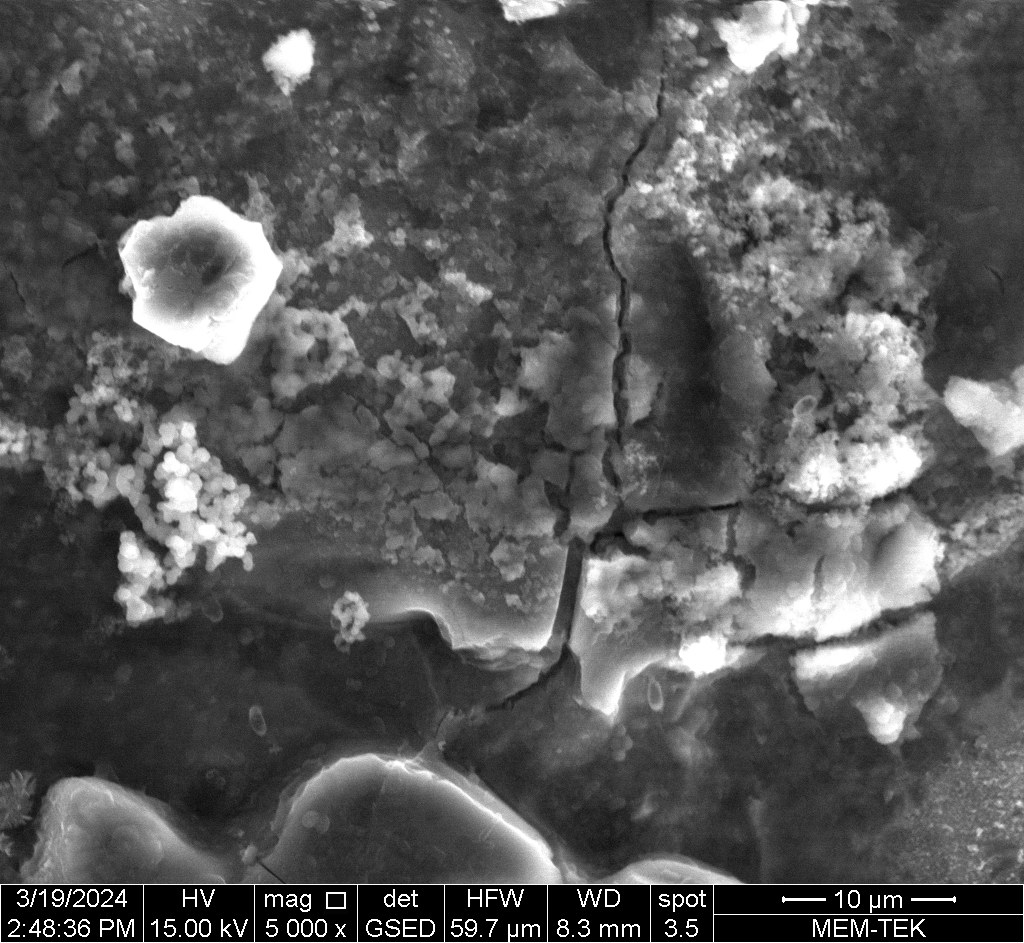

Supplement: Supplementary file 1 [file microorganisms-13-02555-s001.zip › Figure S1. 2024.03.19_SEM/3_5.tif]

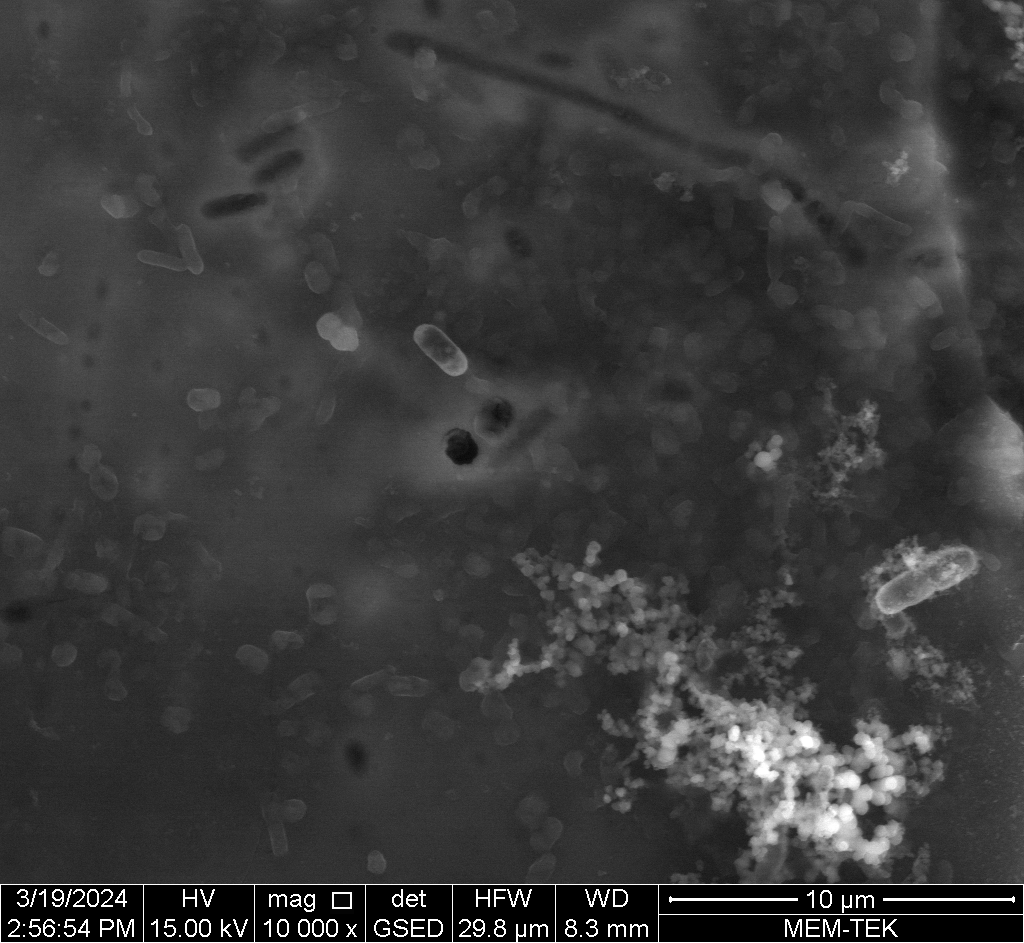

Supplement: Supplementary file 1 [file microorganisms-13-02555-s001.zip › Figure S1. 2024.03.19_SEM/3_6.tif]

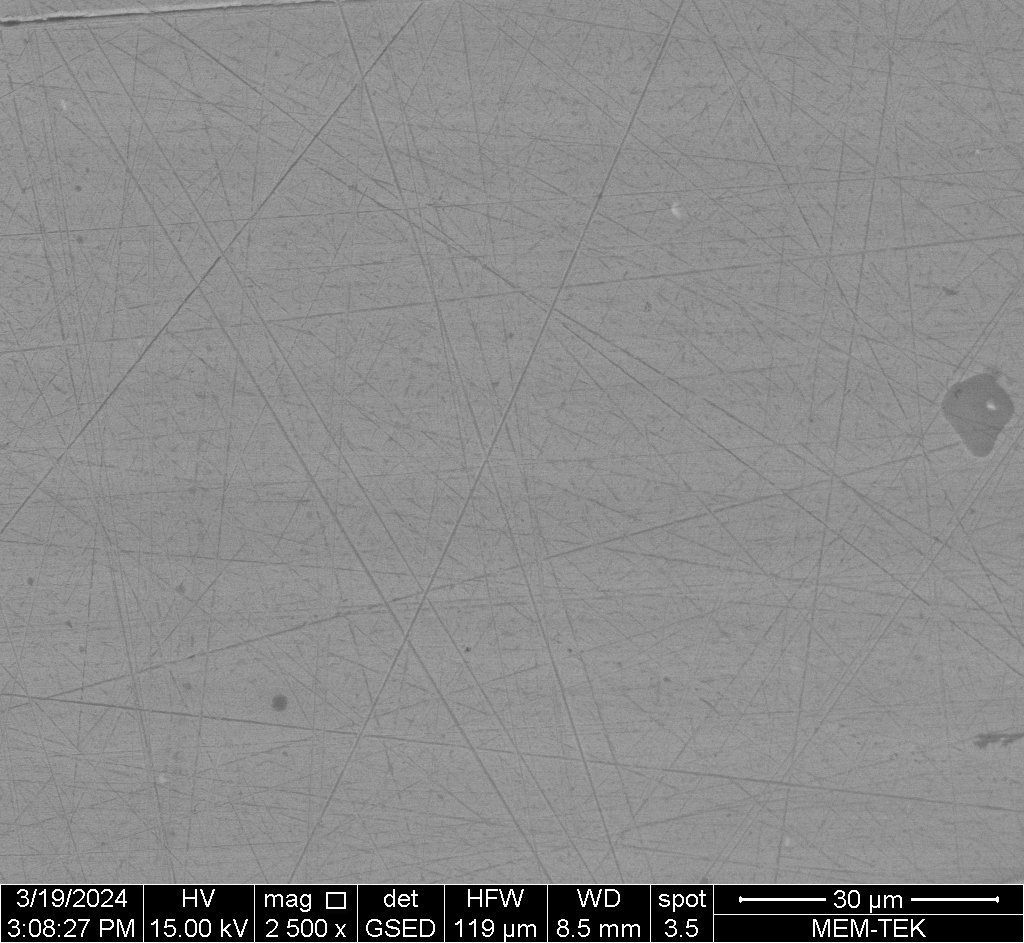

Supplement: Supplementary file 1 [file microorganisms-13-02555-s001.zip › Figure S1. 2024.03.19_SEM/4_1.tif]

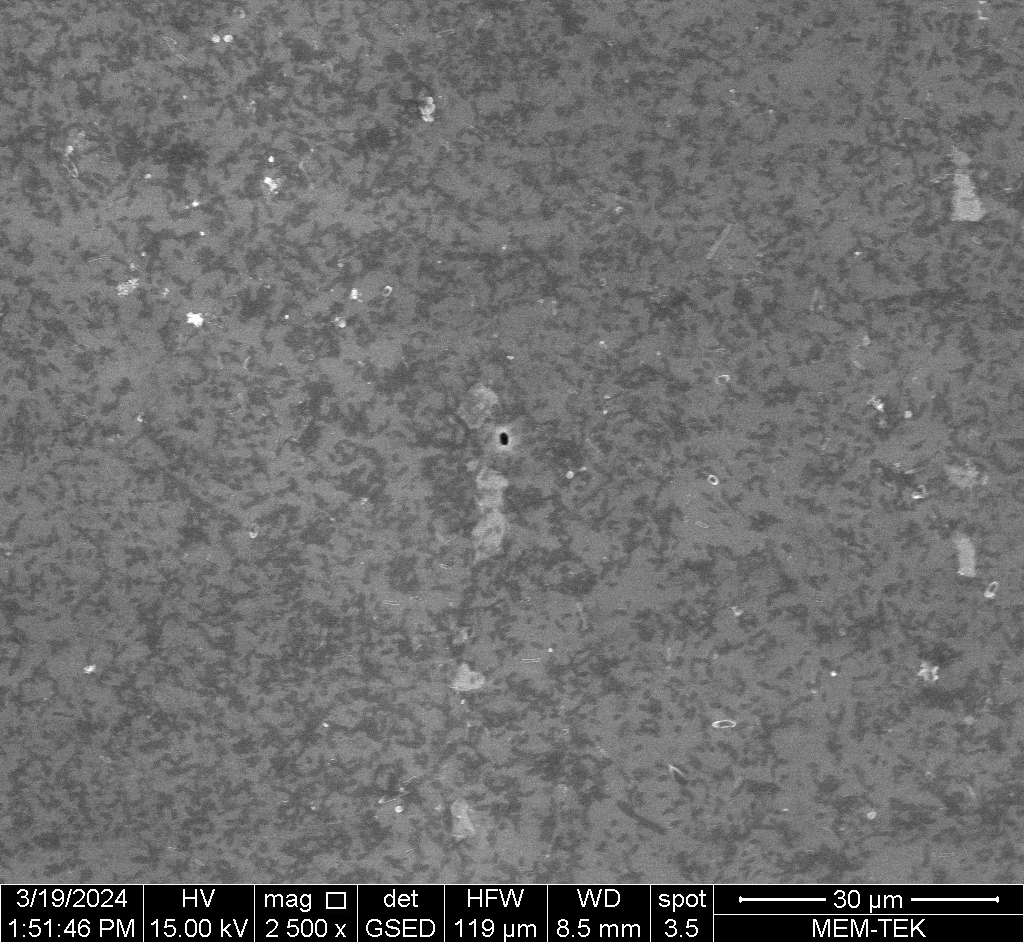

Supplement: Supplementary file 1 [file microorganisms-13-02555-s001.zip › Figure S1. 2024.03.19_SEM/4_2.tif]
